# Longitudinal study of Senecavirus a shedding in sows and piglets on a single United States farm during an outbreak of vesicular disease

**DOI:** 10.1186/s12917-017-1172-7

**Published:** 2017-08-31

**Authors:** Steven J. P. Tousignant, Laura Bruner, Jake Schwartz, Fabio Vannucci, Stephanie Rossow, Douglas G. Marthaler

**Affiliations:** 1 Swine Vet Center, 1608 South Minnesota Avenue, St. Peter, MN 56082 USA; 2 Department of Preventive Medicine, College of Veterinary Medicine, University of Minnesota, 1333 Gortner Avenue, St. Paul, MN 55106 USA

**Keywords:** Swine, Senecavirus a, Seneca Valley virus, Shedding, Epidemiology, Vesicular disease, Case report

## Abstract

**Background:**

The study highlights the shedding pattern of Senecavirus A (SVA) during an outbreak of vesicular disease in a sow farm from the South-central Minnesota, USA. In this study, 34 individual, mixed parity sows with clinical signs of vesicular lesions and 30 individual piglets from 15 individual litters from sows with vesicular lesions were conveniently selected for individual, longitudinal sampling. Serum, tonsil, rectal, and vesicular swabs were collected on day1 post outbreak, and then again at 1, 2, 3, 4, 6, and 9 weeks post outbreak. Samples were tested at the University of Minnesota Veterinary Diagnostic Laboratory for SVA via Real Time Polymerase Chain Reaction (RT-PCR)

**Results:**

In sows, vesicular lesions had the highest concentration of SVA, but had the shortest duration of detection lasting only 2 weeks. Viremia was detected for 1 week post outbreak, and quickly declined thereafter. SVA was detected at approximately the same frequency for both tonsil and rectal swabs with the highest percentage of SVA positive samples detected in the first 6 weeks post outbreak. In suckling piglets, viremia quickly declined 1 week post outbreak and was prevalent in low levels during the first week after weaning (4 weeks post outbreak) and was also detected in piglets that were co-mingled from a SVA negative sow farm. Similar to sows, SVA detection on rectal and tonsil swabs in piglets lasted approximately 6 weeks post outbreak.

**Conclusion:**

The study illustrates the variation of SVA shedding patterns in different sample types over a 9 week period in sows and piglets, and suggests the potential for viral spread between piglets at weaning.

## Background

Senecavirus A (SVA) or commonly known as Seneca Valley Virus belongs to the family *Pircornaviridae* and has been associated with idiopathic vesicular lesions on the snout and coronary bands in swine [3, 8]. In 2014, SVA outbreaks were reported in Brazil and subsequent SVA outbreaks occurred in the United States (US) during the 2015 [2, 4, 5, 11, 12]. While not a new virus to the US or the world, the alarmingly high rate of severe clinical disease anecdotally reported in Brazil and the US, suggests a change in the epidemiology of SVA, such as the emergence of a new variant strain [7, 8, 10]. Some swine farms report reproductive issues, and weak piglets suffering from secondary infections in the nursery [9]. Vesicular disease caused by SVA infection cannot be differentiated from other US Foreign Animal Diseases (FADs), such as foot-and-mouth disease (FMD), swine vesicular disease (SVD), vesicular stomatitis, and vesicular exanthema. Therefore, SVA has important implications in the transport and slaughter of pigs, potentially leading to significant complications with international trade.

In early September of 2015, a sow farm in South-central Minnesota, US, exhibited the sudden and dramatic onset of severe vesicular lesions in nearly 80% of all sows on the site. Due to the relative paucity of information regarding this virus in the field, the objectives of this study were to assess the shedding patterns of SVA in a population of sows and suckling piglets, and assess the potential spread of SVA in weaned pigs. Not only will this information provide valuable insight into the infection dynamics of SVA within a population, but it may also aid in future FADs investigations, as well as assisting in the development of better control and elimination measures of SVA in sow herds.

## Methods

**Fig. 1:**
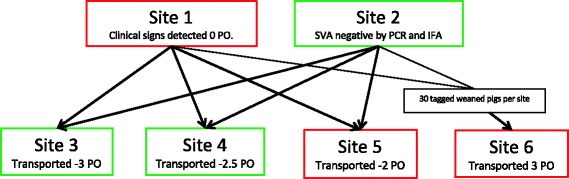
Diagram of the farms (sites 1–6) involved in this study. Color of the box indicates SVA status; green is negative and red is positive. Pigs were transported at different times weeks PO

A farm in South central Minnesota, US, containing two geographically different sow sites (sites 1 and 2, Fig. 1) consisting of 1300 sows per site were selected for this study based on the outbreak of vesicular lesions at one of the sow sites (site 1). This farm maintains ownership of pigs throughout all life stages, and at the time of weaning, piglets are transported from sites 1 and 2 to multiple different weaned pig sites (site 3, 4, 5, and 6) within their company (Fig. 1).

Upon visual detection of vesicular lesions at site 1 (day 0 post outbreak (PO)), a FADs investigation was initiated and samples were collected by the state veterinary office and sent to the National Veterinary Services Laboratories (NVSL), the Foreign Animal Disease Diagnostic Laboratory at Plum Island Animal Disease Center and the University of Minnesota Veterinary Diagnostic Laboratory (UMNVDL). At NVSL, the samples were tested for Foot-and-Mouth Disease virus (FMDv), SVA, and Swine Vesicular Disease virus (SVDv) by real time RT-PCR. At the UMNVDL, the samples were tested for FMDv according the United States Department of Agriculture Standard Operation Procedure (SOP) titled, “Extraction of Total RNA Using a MagMax-96 Total RNA Isolation Kit for the Detection of Classical Swine Fever and Foot-and-Mouth Disease Viruses” (SOP-PVS-0004.1), which is publically available from the NVSL upon request. The detection of SVA occurred by real time Reverse Transcription Polymerase Chain Reaction (RT-PCR) using AgPath-ID One-Step RT-PCR Reagents (Thermo), forward (5′- TCTCTTGCCCTAACACTGGGG-3′) and reverse primers (5′- CTTGCCTCTAAGGACCACCACA-3′) and probe (5′- TGGCCCAAA/ZEN/GTCTCACCACTATGATCAATG-3′) with the following thermal cycling parameters: reverse transcription, 10 min at 45 °C; Taq activation, 10 min at 95 °C; followed by 40 cycles of 15 s at 95 °C, and 45 s at 60 °C.

On the second day post visualization of vesicular disease (day 1 PO), 34 individual, mixed parity sows in gestation from site 1 were conveniently selected based on the presence of vesicular lesions on their snouts and feet. In addition, 30 mixed gender, neonatal piglets from 15-mixed parity litters showing similar clinical signs were conveniently selected and tagged at site 1 between approximately 12 and 24 h of age. Tonsil, rectal, vesicular swabs (when vesicles were present), and serum samples were collected on day 1 and then 1, 2, 3, 4, 6, and 9 weeks PO from both sows and piglets. Last samples were collected at 9 weeks PO and did not occur at weeks 5, 7, and 8 due to concerns of increased stress of the pigs due to sampling. Vesicular swab samples were not collected from the piglets since they lacked clinical vesicular lesions. At week 3 PO (at weaning), the 30 piglets from site 1 were transported to site 6 and were comingled with 30 individually identified mixed gender pigs from site 2 and sampled at 4, 6, and 9 weeks PO (Fig. 1). In addition, at day 0 PO, 10 serum samples and a single oral fluid sample were collected (from three cotton ropes hung in pens within each site) at sites 3, 4, and 5 (Fig. 1) to determine if SVA was present in earlier groups of weaned pigs from before the detection of clinical signs in the system.

Samples were refrigerated overnight and sent next day to the University of Minnesota Veterinary Diagnostic Laboratory. The RNA was extracted using previously described methods [6] for SVA by real time RT-PCR.

## Results

The swine farm initially reported concerns of increased lameness in a pen of group housed replacement gilts, as well as a slight decrease in feed intake (day −2 PO). Day −1 PO, lameness and decreased feed intake was more prevalent among the rest of the sows in the herd. Vesicular lesions were observed on day 0 PO, consisting of un-ruptured vesicles on the snouts and feet of approximately 5–10% of the animals on site 1, and the state veterinary office was notified. By day 2 PO, nearly 80% of the animals in the herd had detectable vesicular lesions (ruptured and un-ruptured) on the snouts and feet. Lameness, lethargy, and decreased feed intake were the main clinical concerns during this time; however, by 1 week PO, there was noticeable improvement in the clinical signs within the population. At no time was fever a major concern within the herd. In farrowing, there was an increase in pre-weaning mortality from an average of 12% to a high of 22% during the first 7 days PO, and returned to average levels by 2 weeks PO. The majority of the piglet mortality was associated with dehydration due to poor milk production by the sow, and piglets being laid on, potentially due to sow lameness. No other clinical signs were detected in piglets in farrowing, and there were no detectable signs at the nursery barns at any point during or after the outbreak.

The samples collected by the state veterinary office at site 1 on the day 0 PO were negative for SVDv at NVSL while the NVSL and UMNVDL had the same results for FMDv and SVA (negative and positive, respectively). Subsequent, SVA testing only occurred at the UMNVDL through the study.

Sows displayed vesicular lesions on the snouts at day 0 PO, and SVA was present in 33 out of 34 (97%) of the vesicle swabs, with an average cycle threshold (Ct) value of 16 (range = 12–19). At 1 week PO, SVA was present in 34 out of 34 (100%) of the lesion swabs. At 2 weeks PO, most lesions had healed (only few skin tags and ruptured vesicles remained in the population), and SVA detection decreased to 12 out of 34 samples (35%). By 3 weeks PO, all of the vesicular lesions had healed, and no additional samples were collected.

**Fig. 2:**
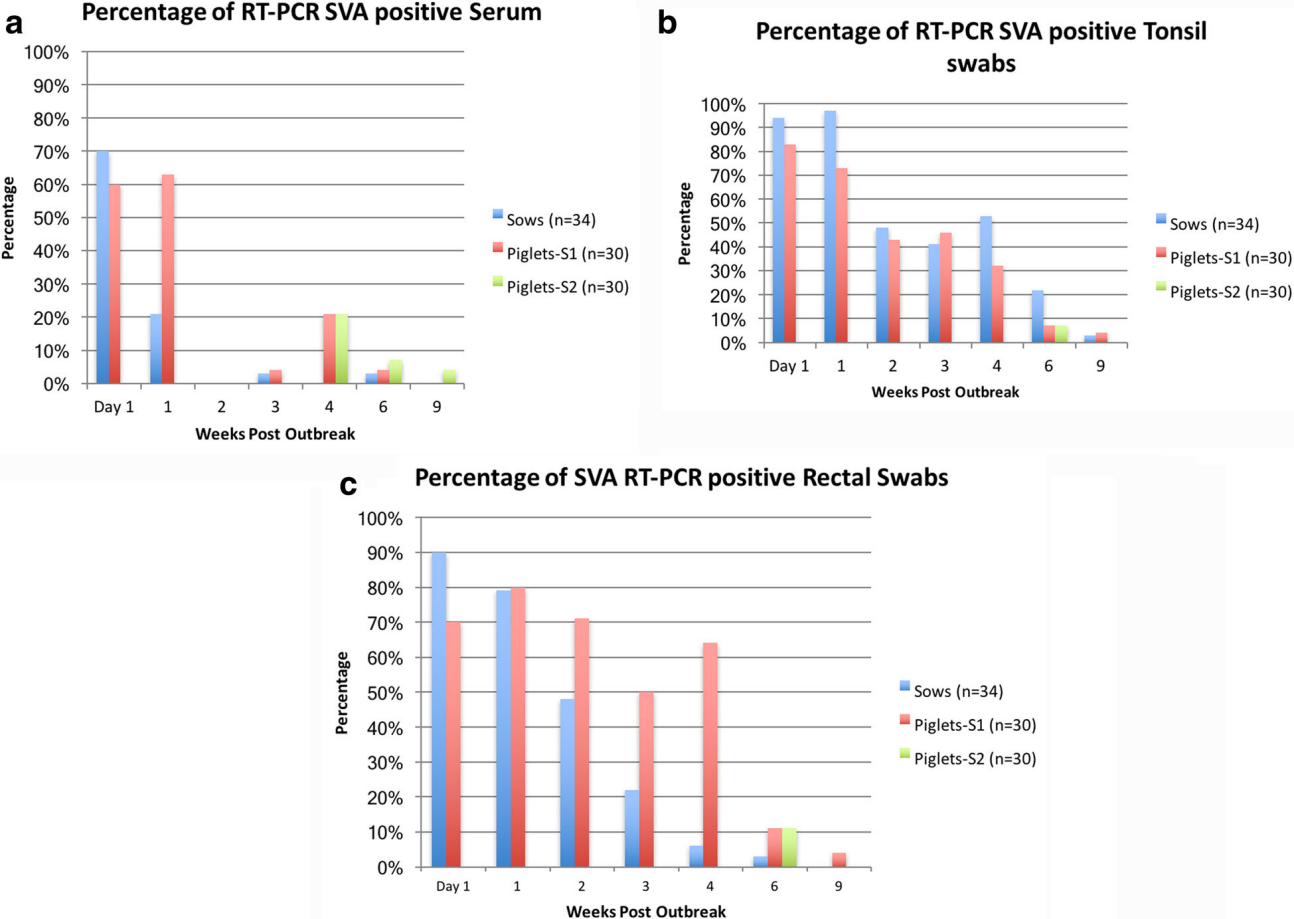
Percentage of serum (**a**), tonsil swabs (**b**), and rectal swabs (**c**) positive for SVA

Generally, viremia was detected up to 1 week PO in sows. A single positive sample was identified at 3 and 6 weeks PO (Fig. 2a). At the first sampling (day 1 PO), the Ct values from the sow serum averaged 33.1 (range = 17–36). Viremia was detected in only 7 out of 34 (20%) of the sows at 1 week PO. SVA viremia was not detected in 11 out of 34 (32%) of the sows at any point during the study. Viremia was detected in 18 out of 30 (60%) and 19 out of 30 (63%) in the suckling piglets from site 1. Additional SVA positive samples were detected at 3, 4, and 6 weeks PO. In suckling piglets, Ct values averaged 30.6 (range = 24–39.8). Similar to sows, viremia was not detected in 9 out of 30 (30%) of the site 1 piglets enrolled in the study. Viremia was detected in site 2 piglets at 4 (6 out of 30 (21%)), 6 (2 out of 30 (7%)), and 9 (1 out of 30 (3%)) weeks PO, with an average Ct value range of 35.7–38.0.

Tonsil swabs yielded the highest percentage of SVA detection in site 1 sows (32 out 34 (94%)) and piglets (25 out of 30 (83%))(Fig. 2b). In sows, the detection of SVA in the tonsils peaked at 1 week PO (33 out of 34 samples (97%)), with average Ct values of 24.8 (range = 20–27). In site 1 piglets, the peak shedding of SVA occurred at day 1 PO, with average Ct values of 27.1 (range = 20.3–34.6). The detection of SVA shedding decreased over time in sows and piglets, and a single sow and piglet tested positive at 9 weeks PO (Ct values of 35 and 36, respectively). SVA was only detected in 2 of the 30 piglets (7%) from site 2 at 5 weeks PO.

The peak of SVA detection from rectal swabs in sows (31 out of 34 samples (91%)) occurred at day 1 PO and continued to steadily decrease and was not detected at 9 weeks PO (Fig. 2c). In site 1 piglets, the detection of SVA peaked at 1 week PO when 27 of the 30 samples (90%) tested positive. While the detection of SVA decreased in site 1 piglets at 2 and 3 weeks PO, 19 out of 30 samples (64%) of the rectal swabs were positive at 4 weeks PO. At 6 weeks PO, the detection of SVA was same for both site 1 and 2 piglets (11%, *n* = 3); however, a single piglet from site 1 was still shedding SVA at 9 weeks PO.

The oral fluid and serum samples were positive for SVA from site 5, but negative from site 3 and 4. SVA was detected in 9 out of 10 (90%) of the serum samples (Ct values averaged 32.8, range = 31.8–35.9), and the single oral fluid sample had a (Ct value = 24.3), despite the fact that there were no obvious detectable clinical signs in site 5. Of importance is that site 5 appears to have received SVA positive weaned pigs from site 1 before the detection of clinical signs of decrease in feed intake and vesicular lesions.

## Discussion

The study assessed the shedding pattern of SVA in sows and piglets during an outbreak on a farm in the US and investigated the spread of SVA between pigs during the post weaning period. In addition, the study suggests the spread of SVA to distant pig sites via piglets before the onset of vesicular lesions.

Fresh vesicular lesions on the sows had the greatest amounts of SVA; however, the lesions were only present for a very short period of time (2 weeks). This is important because state and federal veterinarians conducting the FAD investigations are trained to identify vesicles and collect their fluid. However, these data suggest a lower rate of detection of SVA in ruptured vesicles and dried skin tags (that are present after the first week of the outbreak) when compared to tonsil and rectal swabs, especially later in the outbreak when vesicles were no longer present. In sows, the detection of SVA in tonsil and rectal swabs was greater than 90% at 0 week PO and remained as high as 50% through 5 weeks PO, these sample types should be collected and submitted, in addition to vesicular lesion swabs and fluid (if present), as part of FAD investigations for the detection of SVA. In addition, the detection of SVA in oral fluid samples collected from site 5 and the high percentage of positive SVA tonsillar swabs suggests they may be valuable tools for the detection of SVA in pigs.

Surprisingly, SVA was detected in serum and oral fluids from site 5, which had received weaned pigs approximately −2 weeks PO, before the detection of clinical signs at site 1. Furthermore, obviously detectable clinical signs were lacking in piglets at site 5, indicating the potential to spread SVA without realization through weaned pig movements. This is a major concern for epidemiologists as well as state and federal veterinarians because the prevalence of SVA within the US swine herd could be greatly underestimated. Therefore, as part of any epidemiological investigation of SVA outbreaks (particularly on sow farms), a social network analysis, which details all pig movements to the level of the specific trailers and trucks used during the previous month should be conducted to identify the potential scope of virus spread.

Commingling of piglets at weaning is common in the US swine industry. When the piglets were commingled at week 4 PO (approximately 1 week after weaning), 12 of the 30 (40%) of the piglets sampled from sites 1 and 2 were viremic. Interestingly, site 1 and 2 piglets had the same level SVA shedding in the tonsil and rectal swabs (2 out of 30 (7%), and 3 out of 30 (10%), respectively) at 6 weeks PO. These results suggest the potential spread of SVA to site 2 piglets, and SVA may be transmitted during commingling of piglets. Therefore, caution should be exercised when considering the movement of weaned pigs during an outbreak, which includes avoiding the comingling of piglets from different sow farms.

While SVA was detected in site 2 piglets, the levels of virus were minimal in the tonsil and rectal swabs potentially implying some underlying immunity to SVA in site 2 piglets that limited viral infection and shedding. While site 2 tested negative by PCR, indicating that the herd was not infected at that time, a proper immunological assay, which may help elucidate if the herd had been infected in the past and has some level of immunity, is lacking at the time of writing this manuscript. With that in mind, immunological assays including several different ELISA targets are currently under development at several US veterinary diagnostic laboratories [1]. In addition, these immunological assays would help to understand the prevalence of this virus in the US swine herd (in particular sow herds), which would provide better planning and allocation of resources for future SVA outbreaks that would initiate FAD investigations needed by state and federal veterinarians.

Our results suggest that SVA can be detected in a population of sows in tonsil and rectal swabs for up to 9 weeks; however, the viability of SVA at 9 weeks is currently unknown. Future studies should be developed to discern the viability of the virus from these samples, especially in the later stages of the outbreak. This information would be beneficial to veterinarians working on eliminating SVA from affected sow farms since it would dictate the duration of sow herd closure (no entry of potentially immunologically naïve animals for the duration of the shedding period) in order to elimination the virus from that population. Additionally, as part of any plan to eliminate SVA from sow farms, achieving rapid and homogeneous exposure of all animals is crucial. Therefore, studies are needed to identify the most efficient route of SVA exposure to eliminate the virus from infected sow herds.

## Conclusion

In conclusion, this study highlights the detection of SVA in vesicular lesions, serum, tonsil and rectal swabs and suggests the spread of SVA between piglets during the post weaning period. In addition, this study suggests that tonsil and rectal swabs, and oral fluids may be useful for monitoring populations SVA positive animals over time.

## Abbreviations

CT: Cycle threshold
FAD: Foreign Animal Disease
FMD: Foot-and-mouth disease
NVSL: National veterinary service laboratories
PO: Post outbreak
RT-PCR: Reverse Transcription Polymerase Chain Reaction
SOP: Standard operating procedure
SVA: Senecavirus A
SVD: Swine vesicular disease
US: United States
